# Anti-site-induced diverse diluted magnetism in LiMgPdSb-type CoMnTiSi alloy

**DOI:** 10.1038/srep42034

**Published:** 2017-02-07

**Authors:** T. T. Lin, X. F. Dai, R. K. Guo, Z. X. Cheng, L. Y. Wang, X. T. Wang, G. D. Liu

**Affiliations:** 1 School of Material Sciences and Engineering, Hebei University of Technology, Tianjin, 300130, P. R. China; 2 School of Physics and Electronic Engineering, Chongqing Normal University, Chongqing, 400044, P. R. China; 3 Institute for Superconducting and Electronic Materials, University of Wollongong, North Wollongong, NSW 2500, Australia

## Abstract

The effect of three kinds of anti-site disorder to electronic structure and magnetic properties of the LiMgPdSb-type CoMnTiSi alloy are investigated. It was found the Mn-Ti anti-site disorder can induce the diluted magnetism in CoMnTiSi matrix. The magnetic structure has an oscillation between the ferromagnetic and antiferromagnetic states with the different degree of Mn-Ti anti-site disorder. Two novel characteristics: the diluted antiferromagnetic half-metallicity and the diluted zero-gap half-metallity are found in the different degree range of the Mn-Ti anti-site disorder. The Co-Mn and Co-Ti anti-site disorder have little effect on the magnetic properties. The width of energy gap and the intensity of DOS at the Fermi level can be adjusted by the degree of Co-Mn or Co-Ti anti-site disorder. The independent control to the carrier concentration and magnetization can be realized by introducing the different anti-site disorder.

In the recent decades, Heusler alloys have attracted great interest because many novel materials have been found in these alloys, like half-metal^1^, topological insulator^2^, spin-gapless semiconductor^3^ and spin-gapless half-metal^4^ etc. These materials are essentially due to their exceptional electronic structure, which qualify them as promising functional materials in various spintronic devices, such as giant magnetoresistance spin valves, magnetic tunnel junctions, spin-injecting and spin-transfer torque devices. Another reason to make Heusler alloys become a research hotspot is that Heusler alloys with different functional characteristics have the same crystallographic structure and some of them even are very close in composition and electronic structure^5^,^6^,^7^. This can minimize the mismatching of lattice and electronic structure and remain the excellent performances when materials with different functional characteristics are applied in combination in spintronic devices. In addition, Heusler structure can match well with traditional diamond and zinc-blende structures adopted by a large number of semiconductors because of the similarity of Heusler structure and zinc-blende (diamond) structure.

Diluted magnetic semiconductors (DMS) are a class of materials that can pave the way to develop both the spin and the charge of carriers^8^,^9^ and have huge potential applications in next generation spintronic devices. DMS has a little lattice and electronic structure mismatching to their matrix. At present, there has been a rapidly growing interest in the field of semiconductor and DMS with Heusler structure. Up to now, CoTiSb, NiTiSn, CoNbSn^10^, Fe_2_VAl^11^, Fe_2_TiSn^12^,^13^ and Fe_2_TiSi^14^ with Heusler structure have been reported to be semiconductors. Particularly, the investigations on DMSs, the transition-metal-element-doped CoTiSb alloys, were carried out in a large number^15^,^16^,^17^. The Curie temperature of the Fe-doped CoTiSb alloy is up to 700 K, which is the highest for all the known DMSs^15^. More recently, we present a viewpoint that the diluted magnetism in semiconductor with Heusler structure can be induced by the atomic anti-site disorder between the different crystallographic sites and it is realized in CoFeTiAl alloy^18^. And Cao *et al*. also reported that anti-site disorder leads to ferromagnetism in α-FeSi_2_ thin film by both experiment and theory^19^. The diluted magnetism in semiconductor induced by the anti-site disorder is one of the unique advantages for Heusler alloys. Dismatching problem of semiconductor and spintronic material in multilayer films can be well solved because the anti-site disorder raises little change on crystal structure, lattice parameter and electronic structure. So, the pursuit of anti-site-induced diluted magnetic semiconductor is a very good idea. In this paper, we try to investigate the effect of anti-site disorder on the electronic structure and magnetic properties of LiMgPdSb-type CoMnTiSi alloy which is reported to be a semiconductor in ref. 20. There are two unexpected characteristics in CoMnTiSi alloy with anti-site-induced diluted magnetism: 1) The coexistence of diluted magnetism, the net magnetic moment of zero and 100% spin polarization. The material with the coexistence characteristic was only reported by Akai *et al*. in 2006 and was called as half-metallic diluted antiferromagnetic semiconductor (HMDAFS)^21^. Although HMDAFS is rarely found, this kind of material is a desirable candidate for the application in magnetoelectronic devices since they would not give rise to strong stray fields and therefore is less affected by external magnetic field. 2) The coexistence of diluted magnetism and zero-gap half-metal. The coexistence characteristic is firstly reported in this paper. We propose such a new type of material to be called as diluted magnetic zero-gap half-metal (DMZGHM). Zero-gap half-metal was first proposed by Y. Du *et al*. in Fe_2_CoSi alloy^4^. The electronic structure feature is that the Fermi level lies in an energy gap with the width of zero in one spin channel as well as the Fermi level has a metallic overlapping with the valence bands in the other spin channel. Such a unique band structure may provide many novel properties. In addition, in this paper, we also discuss the effect of different anti-site disorder on the electronic and magnetic structure in details.

## Results and Discussion

**Figure 1:**
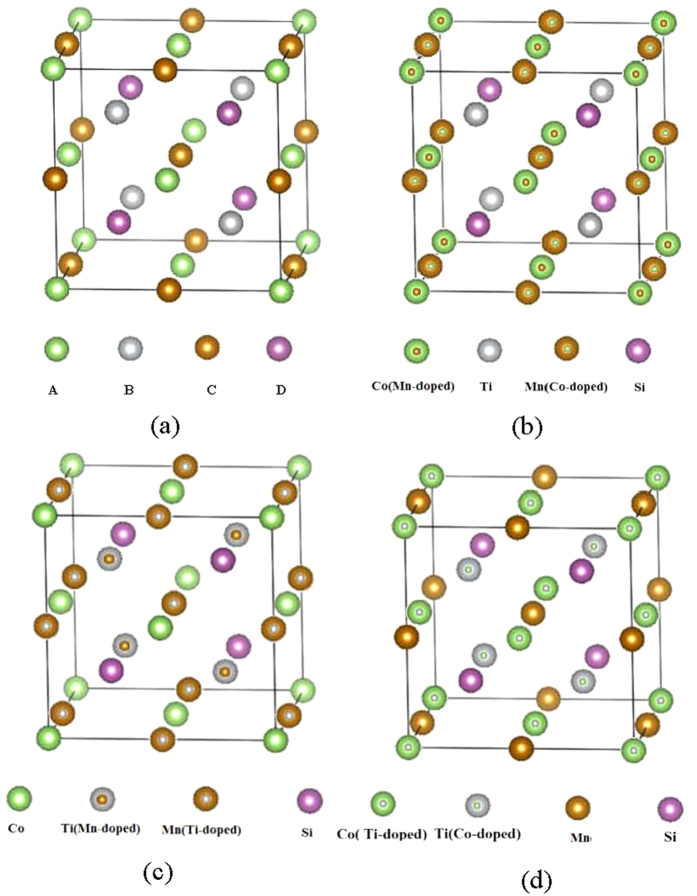
The structure sketches. (**a**) The LiMgPdSb-type structure (quaternary Heusler structure). A(0,0,0), B(0.25,0.25,0.25), C(0.5,0.5,0.5), and D(0.75,0.75,0.75) represent four f.c.c sublattice sites, respectively. (**b**) Co-Mn anti-site disorder, (**c**) Co-Ti anti-site disorder and (**d**) Mn-Ti anti-site disorder.

The LiMgPdSb-type structure(also known as quaternary Heusler structure) can be considered as four interpenetrating f.c.c sublattices, which has four unique crystallographic sites, defined as A(0,0,0), B(0.25,0.25,0.25), C(0.5,0.5,0.5), and D(0.75,0.75,0.75) as shown in Fig. 1(a). For CoMnTiSi compound, it is reported that the ground state (GS) is that the Co, Mn, Ti and Si atoms occupy A, C, B and D sites, respectively, and the completely ordered CoMnTiSi alloy exhibits semiconductivity in its GS. In quaternary Heusler structure, there are three kinds of anti-site disorder between the transition metal atoms: Co-Mn, Co-Ti, and Mn-Ti(The sketches were shown in Fig. 1(b),(c) and (d)). In this paper, we use “x% Co-Mn anti-site disorder” to represent the exchange of x% of the Co atom number at A site and x% of Mn atom number at C site. The same expression is also applied to Co-Ti and Mn-Ti anti-site disorder. In calculations, the equilibrium lattice parameter of 5.73 Å reported in reference^19^ for the completely ordered CoMnTiSi alloy was adopted for all the CoMnTiSi alloys with different anti-site disorder levels and fashions because the level of anti-site disorder investigated in this paper is very low and the effect of anti-site disorder to lattice parameter is very slight.

**Figure 2:**
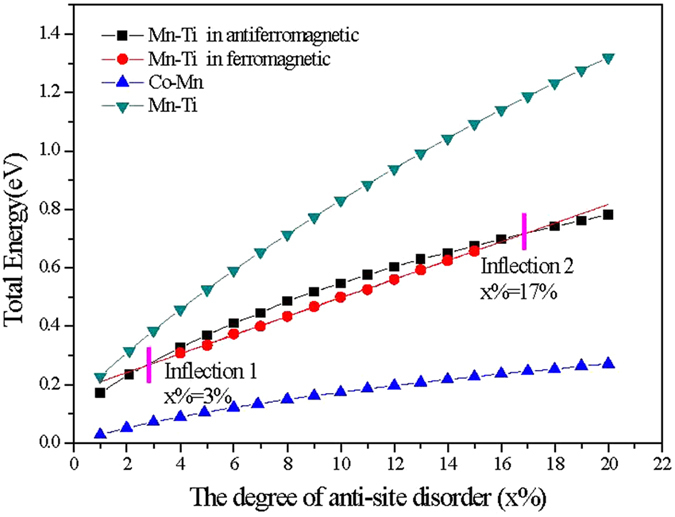
The curves of the total energy dependence to the anti-site disorder degree for the CoMnTiSi alloy with Co-Mn, Co-Ti and Mn-Ti anti-site disorder. The x represents to exchange x% of the atom number at a sublattice site and those at another sublattice site. The total energy of the ordered CoMnTiSi alloy was set as the zero point of Y axis.

Figure 2 shows the total energy dependence relationship to the degree of anti-site disorder for the CoMnTiSi alloys with Co-Mn, Co-Ti and Mn-Ti anti-site disorder. All the three curves show that the total energy nonlinearly and monotonously increases with the increasing degree of anti-site disorder. The Co-Ti anti-site disorder generates the highest incremental intensity in total energy. The total energy of CoMnTiSi alloys with Co-Mn anti-site disorder is always lower than those with the Co-Ti and Mn-Ti anti-site disorder and has an incremental intensity of less than 23.2 meV per 1% anti-site disorder in the whole calculation range. So, it signs that the Co-Mn anti-site disorder is easy to occur and the Co-Ti anti-site disorder needs a much higher energy than Co-Mn anti-site disorder to occur in the CoMnTiSi matrix. The incremental intensity of total energy generated by the Mn-Ti anti-site disorder is in the middle level.

It should be noted that there are two inflections at x = 3 and x = 17 on the curve of the total energy dependence relationship to the degree of Mn-Ti anti-site disorder. These two inflections are corresponding to the transition of magnetic structure, which can be confirmed by the next investigations on density of states (DOS) and magnetic properties. The thresholds of x = 0.3 and 0.7 are due to the use of KKR-CPA in conjunction with the LSDA used in this paper. The use of other methods will reproduce the same trends but probably the exact values of these thresholds would change. A comparison between the results calculated by a full-potential treatment and by KKR-CPA method can be found as Supplementary information. In order to illustrate the relationship between the total energy, magnetic structure and the degree of Mn-Ti anti-site disorder more clearly, the total energy of the ferromagnetic (the magnetic moment of Mn(B) is parallel to that of Ti(C)) and antiferromagnetic (the magnetic moment of Mn(B) is antiparalell to that of Ti(C)) states are respectively plotted in Fig. 2. From Fig. 2, it can be seen that the total energy of the antiferromagnetic state (AFMS) is lower than the ferromagnetic state (FMS) when the degree of Mn-Ti anti-site disorder (x%) is lower than 3%. The FMS have a lower total energy in the range of 3% < x% < 17%. When x% is higher than 17%, the total energy of AFMS returns to a lower value than that of FMS. All of these indicate that the magnetic structure goes through an oscillation between the ferromagnetic and antiferromagnetic states with the different degree of Mn-Ti anti-site disorder. In the range of ferromagnetic GS, the total energy linearly increases with the increasing x and the incremental intensity is 32.0 meV per 1% anti-site disorder.

**Figure 3:**
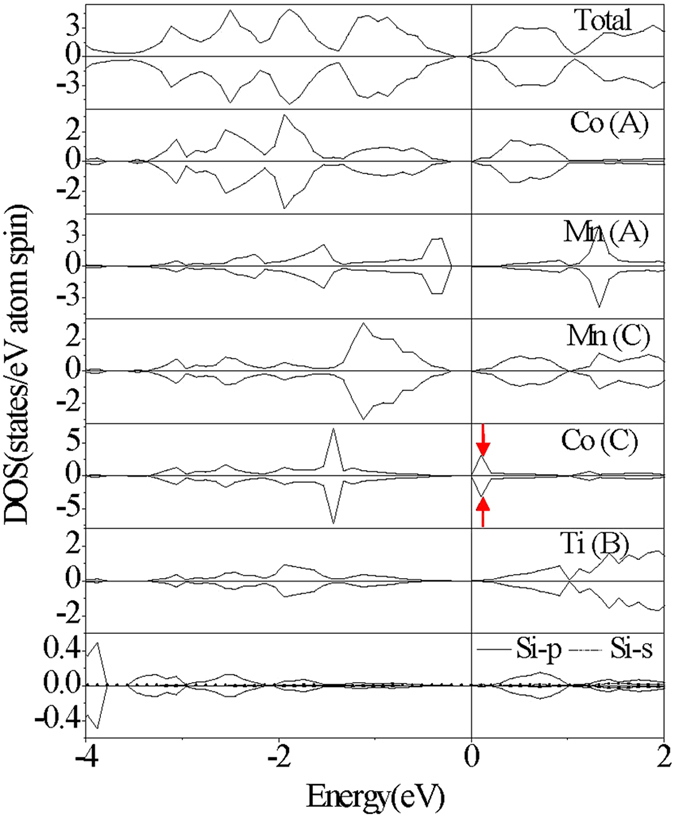
The calculated total DOS and atom-projected PDOS plots for CoMnTiSi alloy with 4% Co-Mn anti-site disorder. What the red arrows denote is antibonding states generated by the hybridization of Co(C) at the anti-site and Co(A) at the normal site.

**Figure 4:**
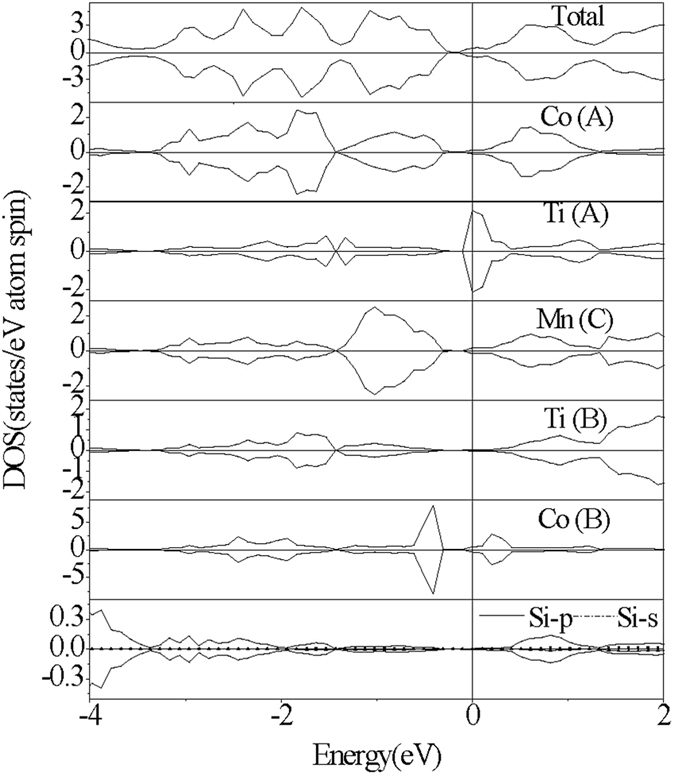
The calculated total DOS and atom-projected PDOS plots for CoMnTiSi alloy with 4% Co-Ti anti-site disorder.

**Figure 5:**
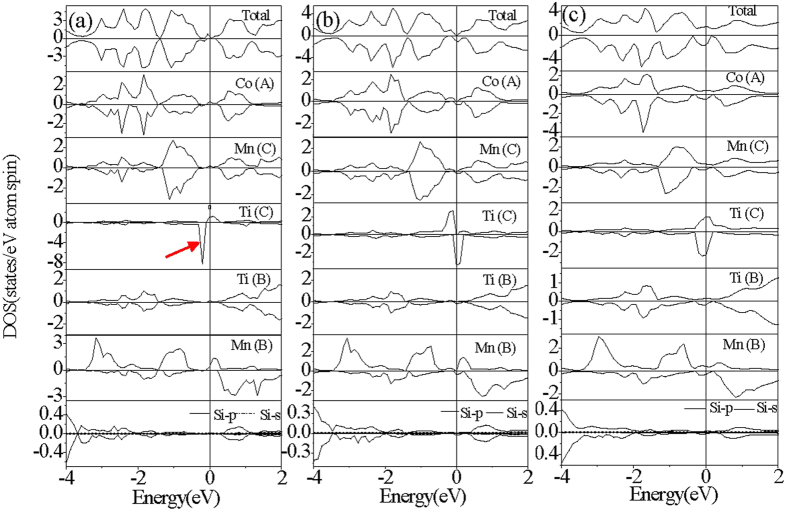
The calculated total DOS and atom-projected PDOS plots for CoMnTiSi alloy with (**a**) 2%, (**b**) 8%, (**c**) 20% Mn-Ti anti-site disorder. What the red arrow denotes is the sharp DOS peak of Ti(C) atom at the anti-site.

The electronic structures of the CoMnTiSi alloy with three kinds of anti-site disorder are calculated. Several typical patterns of the total density of states(TDOS) are shown in Figs 3 and 5. From Figs 3 and 4, it can be seen that Co-Mn and Co-Ti anti-site disorder do not induce any spin splitting and have little effect on the magnetic properties. The Co-Mn anti-site disorder induces the DOS peaks above the Fermi level, which decreases the width of energy gap from 0.22 eV to 0.11 eV. From the atom-projected PDOS shown in Fig. 3, it can be seen that the induced DOS peaks by Co-Mn anti-site disorder above the Fermi level mainly originate from the antibonding states (denoted by red arrows in Fig. 3) generated by the hybridization of Co(C) at the anti-site and Co(A) at the normal site. The Co-Ti anti-site disorder also induces the DOS peaks and the difference from Co-Mn anti-site disorder is that the induced DOS peaks fill in the energy gap of the CoMnTiSi matrix and make the energy gap disappear. The Femi level crosses a DOS peak induced by Co-Ti anti-site disorder. So, the Co-Ti anti-site disorder has a great effect on the carrier concentration but little effect on the magnetic properties. The atom-projected PDOS patterns are also plotted in Fig. 4 for the CoMnTiSi alloys with Co-Ti anti-site disorder. It is clear that Ti(A) and Co(B) atoms at the anti-site have the main contribution to the DOS in the energy gap of the CoMnTiSi matrix. The Mn(C) an Co(A) at the normal site also have a little contribution to the DOS in the energy gap of the CoMnTiSi matrix for they hybridized with the atoms at the anti-site.

The effect of Mn-Ti anti-site disorder on the electronic structure and magnetic properties is more complex. Figs 5(a–c) show the typical DOS patterns of the GS for the CoMnTiSi alloys with 2%, 8 and 20% Mn-Ti anti-site disorder. These three DOS patterns represent the cases in the ranges of x% < 3%, 3% < x% < 17% and x% > 17%, respectively. It can be seen that, for these three cases, the spin-up and spin-down DOS configurations are not as symmetrical as the completely ordered CoMnTiSi alloy, which indicates that Mn-Ti anti-site disorder induces an occurrence of spin splitting and the CoMnTiSi alloys with Mn-Ti anti-site disorder should be in magnetic GS. The critical points of 3 and 17% are corresponding to the inflections on the curve of the total energy dependence relationship to the Mn-Ti anti-site disorder degree as shown in Fig. 2. From the atom-projected DOS (as shown in Fig. 5(a) and (c)), it is clear that the magnetic moment of Mn(B) at the anti-site is antiparalell to that of Ti(C) at the anti-site, which indicates that the GS of CoMnTiSi alloys with Mn-Ti anti-site disorder is an antiferromagnetic state when x% is less than 3% or higher than 17%. When x is in the range of 3~17%(Fig. 5(b)), all the atomic magnetic moments are in parallel arrangement indicating that the GS is a ferromagnetic one. That is to say, the inflections on the curve of the total energy dependence relationship to the Mn-Ti anti-site disorder degree are the critical points of magnetic structural transition. However, it should be noted that the DOS peaks induced by Mn-Ti anti-site disorder have a different distribution in energy with the different range of Mn-Ti anti-site disorder degree. This indicates that the atomic spin-splitting degree induced by Mn-Ti anti-site disorder is different with the different Mn-Ti anti-site disorder degree.

Next, we discuss the electronic structures and magnetic properties of CoMnTiSi alloys with Mn-Ti anti-site disorder in detail according to the ranges: x% < 3%, 3% < x% < 17% and x% > 17%, respectively. For the cases of x% < 3% (Fig. 5(a)), the GS is AFMS. From Fig. 5(a), it can be seen that an impurity DOS peak induced by Mn-Ti anti-site disorder fills in the energy gap of CoMnTiSi matrix in spin-up channel as well as an small impurity DOS peak partly encroaches on the energy gap below Fermi level in spin-down channel. So, the energy gap disappears in spin-up channel and decreases from 0.22 to 0.20 eV in spin-down channel. It should be noted that the Fermi level just lies in the energy gap in spin-down channel and on the impurity DOS peak induced by Mn-Ti anti-site disorder in spin-up channel, which indicates that the CoMnTiSi alloys with Mn-Ti anti-site disorder show a half-metallicity and the spin polarization is up to 100% at the Fermi level when they are in antiferromagnetic ground state (x% < 3%). The atom-projected PDOS patterns are also plotted in Fig. 5(a) for the CoMnTiSi alloys with Mn-Ti anti-site disorder (x% < 3%). It can be seen that the DOS in the spin-up energy gap of the CoMnTiSi matrix is mainly composed of the antibonding DOS of Mn(A) and Ti(C) atoms at the anti-site. The Mn(C) an Ti(B) at the normal site also have a little contribution to the DOS in the energy gap of the CoMnTiSi matrix for they hybridized with the atoms at the anti-site. In spin-down channel, the DOS encroaching on the energy gap below Fermi level comes from the sharp DOS peak (denoted by red arrows in Fig. 5(a)) of Ti(C) atom at the anti-site. The sharp DOS peak has hardly any hybridization with the other atoms and mainly located by the atomic spin-splitting originating from intra-exchange interaction (Hund’s rule).

**Figure 6:**
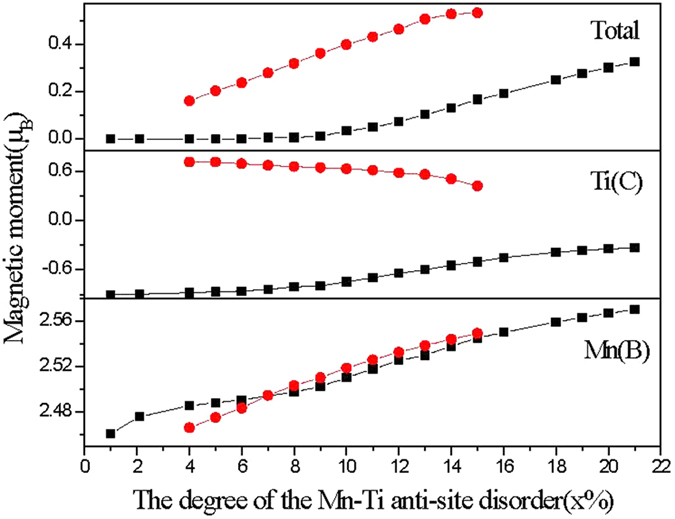
The dependence of the total and atomic magnetic moments to the Mn-Ti anti-site disorder degree for CoMnTiSi alloys with Mn-Ti anti-site disorder. The red symbol + line is the dependence of magnetic moment in ferromagnetic state and the black symbol + line in antiferromagnetic state.

Furthermore, from the atom-projected PDOS, we also can see that the Mn(B) and Ti(C) atoms at the anti-site have a large spin splitting and the magnetic moment of Mn(C) is antiparalell to that of Ti(C). The Mn(B) and Ti(C) atoms are the main contributors of the magnetization and the other atoms have little spin splitting but a very small magnetic moment for the hybridization between the atoms at anti-sites and normal sites leads to a small amount of DOS transfer. The total and atomic magnetic moments were gathered in Fig. 6 and the magnetic moment dependences to the anti-site disorder degree were plotted in this figure. From Fig. 6, it can be observed that the total magnetic moment of CoMnTiSi with the Mn-Ti anti-site disorder is zero when the x% is lower than 3%. So, the CoMnTiSi alloys with Mn-Ti anti-site disorder are the diluted antiferromagnetic half-metals when the Mn-Ti anti-site disorder degree is lower than 3%. That is to say, they have a net magnetic moment of zero, 100% conductive electron spin-polarization and diluted antiferromagnetism. The spin splitting of the Mn(B) and Ti(C) atoms slightly increases with the increasing Mn-Ti anti-site disorder degree when x% is lower than 3%. At the same time, the total magnetic moment is always zero due to the antiparalell arrangement of atomic magnetic moments to counteract their increments. The diluted antiferromagnetic half-metallic DOS feature can be kept in the whole range of x% < 3%.

When the Mn-Ti anti-site disorder degree is in the range of 3% < x% < 17%, the FMS is the GS. From Fig. 5(b), it is clear that the Fermi level lies in a energy gap with the width of zero eV in spin-up channel and has a metallic overlapping with valence bands in spin-down channel. This especial electronic structure at Fermi level is similar to the so-called zero-gap half-metal which is firstly reported and called in Fe_2_CoSi Heusler alloy.[4] However, we should note that the Fe_2_CoSi is a classical ferromagnet but not a diluted magnetic semiconductor which is different from the reported materials in this paper where the especial electronic structure at the Fermi level mainly originates from the impurity bands induced by Mn and Ti atoms at anti-site. The Mn and Ti atoms at anti-site are dilutedly distributed in the CoMnTiSi matrix. So, the CoMnTiSi alloys are a diluted magnetic semiconductor with the zero-gap half-metallicity when the Mn-Ti anti-site disorder degree is in the range of 3% < x% < 17%. Here, we call these materials as a diluted magnetic zero-gap half-metal (DM-ZGH).

In detail, from Fig. 5(b), it can be seen that in spin-up channel, two DOS peaks induced by Mn-Ti anti-site disorder respectively encroach on the energy gap of matrix from the higher and lower energy, which leads to the energy gap width of zero eV. With the atom-projected PDOS, it can be seen that the DOS peak encroaching on the energy gap from the lower energy mainly originates from Ti(C) atom and that from the lower energy originates from the Mn(B) atom. The Mn(C) and Ti(B) atoms at the normal site also have a contribution to these two DOS peaks. In spin-down channel, the impurity DOS peak mainly originating from Ti(C) atom encroaches on the energy gap from the higher energy. Although a narrow energy gap is still kept in spin-down channel, it is in the lower energy far from the Fermi level. The Fermi level lies on a DOS peak originating from Ti(C) atom in spin-down channel.

In Fig. 6, the total and the atomic magnetic moments were also shown for the cases with 3% < x% < 17%. It is clear that the magnetic moment of Ti(C) at the anti-site decreases with the increasing Mn-Ti anti-site disorder degree as well as the magnetic moment of Mn(B) atom increases. With the help of the atom-projected PDOS, it can be seen that the atomic spin splitting weakens with the increasing Mn-Ti anti-site disorder degree when x is in the range of 3%~17%. At the same time, the hybridization between the atoms at the anti-site and normal site increases with the increasing Mn-Ti anti-site disorder degree, which leads to the increase of DOS transfer and then the reduced magnetic moment of Ti(C) and the enlarged one of Mn(B). Ti(C) and Mn(B) atoms are still the main contributors of the magnetization in the CoMnTiSi alloys when the Mn-Ti anti-site disorder is in the range of 3% < x% < 17%. The total magnetic moment is higher than the cases with x < 3% due to the parallel arrangement between the magnetic moments of Ti(C) and Mn(B) and the increasing anti-site disorder degree. The diluted magnetic zero-gap half-metallity can be kept in the whole range of 3% < x% < 17%.

As to the CoMnTiSi alloys with higher Mn-Ti disorder degree than 17%, the typical DOS and atom-projected DOS patterns are shown in Fig. 5(c). From the Fig. 5(c), it can be seen that a great amount of DOS fills in the energy gap of the CoMnTiSi matrix whether in spin-up or spin-down channel. These CoMnTiSi alloys with Mn-Ti anti-site disorder lose the semiconductivity and become an ordinary ferromagnets without a half-metallicity. Furthermore, from the PDOS patterns, it can be seen that a lot of DOS filled in the energy gap of CoMnTiSi matrix come from the atoms at the normal sites besides the atoms at the anti-site which indicates that the anti-site disorder arouse the electronic structure to change for all the atoms in CoMnTiSi matrix when x% is higher than 17%. The spin splitting is weaker for Ti(C) atoms and larger for Mn(B) atoms when x% > 17% than x% < 3% though the CoMnTiSi alloy are AFMS both in these two ranges of Mn-Ti anti-site disorder degree. Correspondingly, the magnetic moments and their dependence to the Mn-Ti disorder degree are also shown Fig. 6 for the cases with x% > 17%. It can be seen that the magnetic moment of Ti(C) at the anti-site is smaller than that when x is less than 3% and decreases with the increasing anti-site disorder degree as well as the magnetic moment of Mn(B) at the anti-site is larger and increases with the increasing anti-site disorder degree. So, the total magnetic moment increases with the increasing anti-site disorder degree and is not zero as the cases with x% < 3% though the CoMnTiSi is still AFMS when x% > 17%. In addition, it should be noted that all the atoms in CoMnTiSi matrix were affected due to the occurrence of a lot of Mn-Ti anti-site disorder and all the transition metal atoms at the normal sites also show an obvious spin splitting. So, the CoMnTiSi alloys gradually lose the diluted magnetism and become the real ordinary ferromagnets with the increasing Mn-Ti anti-site disorder degree.

## Conclusions

The effect of Co-Mn, Co-Ti and Mn-Ti anti-site disorder to the electronic structure and magnetic properties of CoMnTiSi matrix are systematically investigated. It was found that the width of energy gap and the intensity of DOS at the Fermi level can be adjusted by the Co-Mn or Co-Ti anti-site disorder degree. The Mn-Ti anti-site disorder can induce the diluted magnetism in CoMnTiSi matrix. The independent control to the carrier concentration and magnetization can be realized by introducing the different anti-site disordering. Especially, in this paper, it is first to report the diluted zero-gap half-metal and the diluted antiferromagnetic half-metal with the vanish of net magnetic moment in Heusler alloys. The magnetic structure has an oscillation between the ferromagnetic and antiferromagnetic states with the increasing Mn-Ti anti-site disorder degree. The HMDAFS and DMZGHM occur in different magnetic structure, respectively, and can be kept in a wide range of Mn-Ti anti-site disorder degree. The oscillation of magnetic structure between the ferromagnetic and antiferromagnetic states with the increasing Mn-Ti anti-site disorder degree implies that a possibility to have a nonlinear magnetic behavior near the magnetic structure transition point which is quite worthy of further investigation but it is out of the scope of this paper. Recently, a new experiment to adjust the atomic occupying site was reported^22^. The experimental results shown that He^+^ ion irradiation is an effective method to selectively induce the Co-Mn anti-site disorder in Heusler alloy Co_2_MnSi. This is a good forerunning to selectively induce different anti-site disorder in Heusler alloys.

## Methods

Electronic structure calculations on the CoMnTiSi alloys with various anti-site disorder were performed using the charge self-consistent Korringa–Kohn–Rostoker (KKR) method. The coherent potential approximation(CPA) was employed to deal with the anti-site disorder in the calculations^23^,^24^,^25^,^26^,^27^. The calculations were preformed within the atomic-sphere approximation for the charge density. The crystal potential of muffin-tin form was constructed within the local density approximation (LDA)^28^,^29^. We firstly do test using the different number of K-points from less to more to ensure convergence of the calculations with respect to the number of K-points. Finally, the KKR-CPA Green function was calculated on a 65 special k-point mesh in the irreducible part of the Brillouin zone. The convergence tolerance is 0.001 eV for the total energy. The muffin-tin sphere radii of 2.3 a.u. were used for all the atoms. The density of states was achieved by the tetrahedral integration method^30^.

## Additional Information

**How to cite this article**: Lin, T. T. *et al*. Anti-site-induced diverse diluted magnetism in LiMgPdSb-type CoMnTiSi alloy. *Sci. Rep.* **7**, 42034; doi: 10.1038/srep42034 (2017).

**Publisher's note:** Springer Nature remains neutral with regard to jurisdictional claims in published maps and institutional affiliations.

## Supplementary Material

Supplementary Information
