# Supplementary material for: Anti-site-induced diverse diluted magnetism in LiMgPdSb-type CoMnTiSi alloy
Source: Sci Rep. 2017 Feb 7;7:42034. doi: 10.1038/srep42034 (PMC5294629; doi:10.1038/srep42034)
Supplement: Supplementary Information [file srep42034-s1.pdf]

Anti-site-induced diverse diluted magnetism in LiMgPdSb-type CoMnTiSi alloy

T.T. Lin[1,2], X. F. Dai[1], R. K Guo[1], Z. X. Cheng[3], L. Y. Wang[1], X. T. Wang[1,3] and G. D. Liu[1,2,*]

1 School of Material Sciences and Engineering, Hebei University of Technology, Tianjin, 300130, PR China

2School of Physics and Electronic Engineering, Chongqing Normal University, Chongqing, 400044, PR China

3 Institute for Superconducting and Electronic Materials，University of Wollongong, North Wollongong，NSW 2500, Australia

*Corresponding author. Tel.:86-022-60204765, Email address: gdliu1978@126.com (G. D. Liu)

Supplementary information

The WIEN2K code (where the full-potential linearized augmented plane-wave (FP-LAPW) method is adopted) was employed to calculate the magnetic moment, the DOS and band structures for four atomic arrangement fashions. The calculated results are shown in the Fig.1 and table 1. The corresponding calculated results by KKR-CPA are also put in the same figure and table to contrast. It is clear that the DOS and band structures of KKR-CPA are quite consistent with the results of WIEN2K. Furthermore, quantitatively, the half-metallic gap calculated by KKR-CPA is slightly smaller than that by WIEN2K, which indicates that the KKR-CPA method is more rigor to tell a half-metallicity. The results obtained using the two methods are consistent for the total magnetic moment and magnetic structure. There are some differences in the atomic magnetic moment for these two methods, which can be attributed to the different electron population analysis method. The electron population analysis method only have an effect to atomic magnetic moment and no effect to the DOS and band structures. So, we think that the KKR-CPA method does work to CoMnTiSi alloy and the results calculated by KKR-CPA method are reliable. In addition, we also list several references where the KKR-CPA method is also successfully used to

investigate the DOS, magnetic structure etc. of some Heusler alloys.

As the reviewers pointed out, the critical issues are the appropriateness of the atomic sphere approximation for the semi-metals, and the reliability of the anti-site formation energy absent lattice relaxation. We attempt to address these issues by comparing the DOS, band structure, and magnetic moments of the alloys without the anti-site defects calculated from KKR to the results calculated from FLAPW. The agreement between the two methods indicates that the error due to the ASA is not severe. However, this comparison does not address the issue of lattice relaxation. In fact, the reason that no relaxation needs to be considered in the CPA treatment of disorder is because this is a single-site theory, where lattice relaxation around a defect site is meaningless. Accounting for such relaxation amounts to including site-site correlation which requires one to go beyond the single-site approximation.

we agree with the reviewer's viewpoint that ASA and no relaxation are not critical shortcomings, charge transfer could be. CPA is known for causing large errors in alloys with significant charge transfer. Here, in table 2, we show the amount of charge transfer. The results show the amount of charge transfer is quite small for all the atoms, which supports the correctness of the CPA treatment of the anti-site defect for CoMnTiSi.

Acknowledgements:

We are very grateful to the reviewers for their patience and high level discussions. Especially, for reviewer 3, we solved a lot of critical problems according to his advice and discussion.

Figure captions

Fig. 1. The total DOS and band structure patterns of LiMgPdSb-type CoMnTiSi alloy with four atomic arrangement fashions achieved by KKR-CPA and FP-LAPW method, respectively. The total DOS patters are in left column and the band structure patterns in the right one. CoMnTiSi, MnCoTiSi, CoTiMnSi and TiMnCoSi on the graphs represent the atomic arrangement fashions. The abbreviations, KKR-CPA and FPLAW on the graph, represent that the results on the corresponding graph are achieved by KKR-CPA and FP-LAPW method.

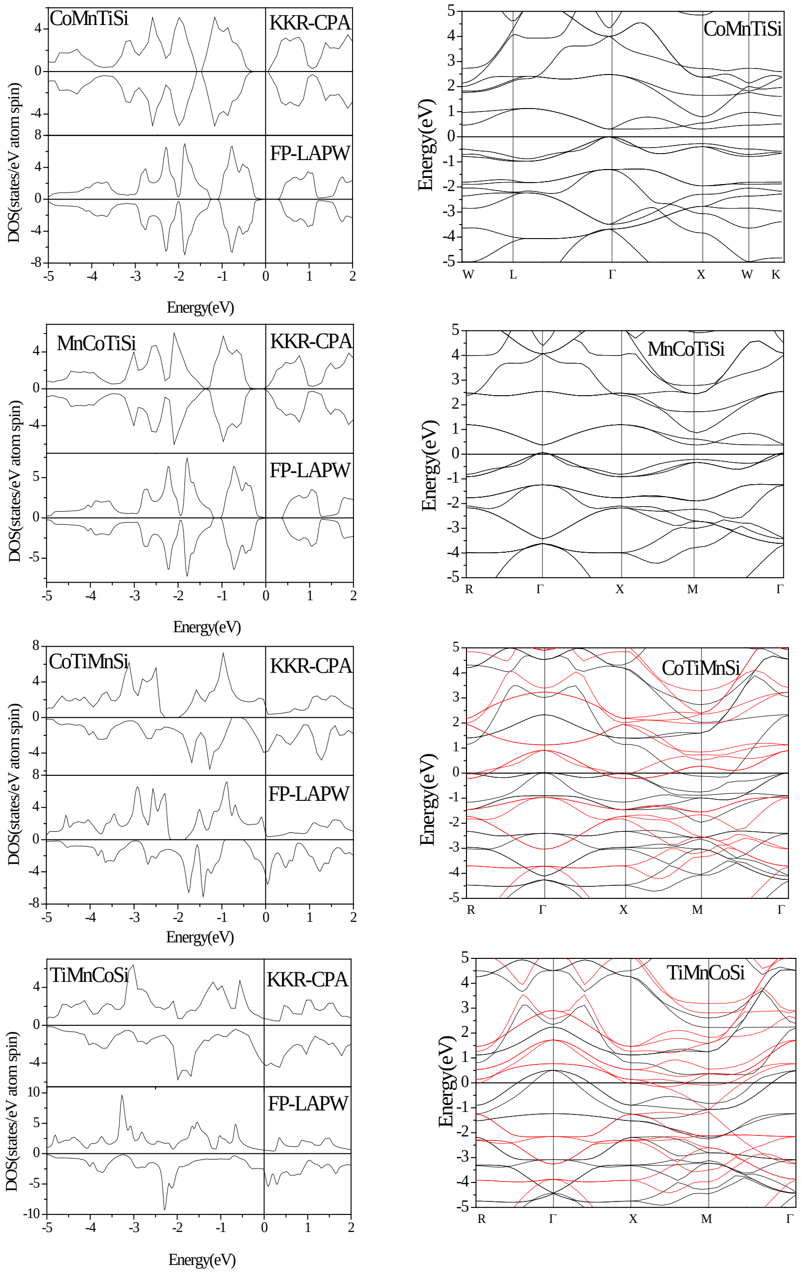
CoMnTiSi
KKR-CPA
FP-LAPW
DOS(states/eV atom spin)
Energy(eV)
W L Γ X W K
MnCoTiSi
R Γ X M Γ
CoTiMnSi
TiMnCoSi

Fig.1

Table Ⅰ. The calculated total magnetic moment (M_{Total}) and atomic magnetic moments by KKR-CPA and FP-LAPW method, respectively. The data of CoMnTiSi can be seen in the formal writting.

Alloys	Method	$M_{Total}(\mu_B)$	Co atom(μ_B)	Mn atom(μ_B)	Ti atom(μ_B)
MnCoTiSi	KKR-CPA	0	0	0	0
	FP-LAPW	0	0	0	0
CoTiMnSi	KKR-CPA	4.05	1.29	2.46	0.21
	FP-LAPW	4.05	1.77	2.719	0.09
TiMnCoSi	KKR-CPA	3.45	1.26	2.16	-0.02
	FP-LAPW	3.62	1.49923	2.16031	-0.07

TableⅡ. The calculated amount of charge transfer for each atom in CoMnTiSi alloy. The 3.125% and 6.25% are the Mn-Ti disordering degree in CoMnTiSi alloy.

Atom / Disorder degree	Co	Mn	Ti (at anti-site)	Ti	Mn (at anti-site)	Si
3.125%	-0.46<br>-0.47<br>-0.41<br>-0.39<br>-0.48<br>-0.42	-0.10<br>-0.12<br>-0.11	-0.05	0.56<br>0.54<br>0.55<br>0.51	0.26	0.02
6.25%	-0.43<br>-0.45<br>-0.42<br>-0.47<br>-0.38	-0.10<br>-0.09<br>-0.11	-0.16<br>-0.17	0.54<br>0.55<br>0.53	0.34<br>0.33	0<br>0.01<br>0.02
